# Representation of Women in Authorship and Dissemination of Analyses of Physician Compensation

**DOI:** 10.1001/jamanetworkopen.2020.1330

**Published:** 2020-03-20

**Authors:** Allison R. Larson, Kelly A. Cawcutt, Meridith J. Englander, Susan C. Pitt, Eman Ansari, Howard Y. Liu, Julie K. Silver

**Affiliations:** 1 Department of Dermatology, Boston University School of Medicine, Boston, Massachusetts; 2 Division of Infectious Diseases, Department of Medicine, University of Nebraska Medical Center, Omaha; 3 Division Pulmonary and Critical Care Medicine, Department of Medicine, University of Nebraska Medical Center, Omaha; 4 Department of Radiology, Albany Medical College, Albany, New York; 5 Department of Surgery, University of Wisconsin School of Medicine and Public Health, Madison; 6 Department of Pediatrics, Harvard Medical School, Boston, Massachusetts; 7 Division of Emergency Medicine, Department of Medicine, Boston Children’s Hospital, Boston, Massachusetts; 8 Department of Psychiatry, University of Nebraska Medical Center, Omaha; 9 Department of Physical Medicine and Rehabilitation, Harvard Medical School, Boston, Massachusetts; 10 Massachusetts General Hospital, Boston; 11 Spaulding Rehabilitation Hospital, Boston, Massachusetts; 12 Brigham and Women’s Hospital, Boston, Massachusetts

## Abstract

This cross-sectional study examines the representation of women in authorship and dissemination of analyses of physician compensation.

## Introduction

Physician gender pay gaps persist in the US despite an impressive body of research spanning more than 25 years and adjusting for potentially confounding factors, including rank, years in practice, practice type, specialty, parental status, and hours worked.^1,2,3,4,5^ We hypothesized that women physicians were disproportionately represented as producers and disseminators of pay equity research and were largely unfunded for this work. Men have great power to drive change, given their larger representation within academic medical leadership. If compensation studies are unfunded and if women are more engaged than men in the pay equity issue, these factors may contribute to slow progress in addressing compensation disparities.

## Methods

This cross-sectional study was performed on US-based physician compensation studies published from January 1, 2013, to February 22, 2019, in refereed medical journals indexed by PubMed. Because this study did not involve human participants and data were publicly available, the Boston University institutional review board determined that review was not required. This study is reported following the Consolidated Standards of Reporting Trials (CONSORT) reporting guideline.

Search details included text word and Medical Subject Headings searches on *salary*, *research support*, *pay*, *compensation*, *wage*, *payment*, and *funding*; *physicians* and *faculty*; and *sex factors*, *gender*, *male*, *female*, *men*, and *women*. References were reviewed in included studies to identify additional related reports. Studies that did not include physician compensation or gender data or were secondary sources (eg, reviews, perspectives) were excluded.

Unique authors in each category were recorded, as were presence and sources of funding for each compensation study. Journal article citations for the studies were collected from the Scopus database (Elsevier) (eAppendix in the Supplement). We ascertained the gender of most authors from their online profiles using their stated pronouns and/or photographs. For authors for whom this was not possible (2.8% of compensation authors, 3.2% of citation authors, 6.8% of disseminators), data were included if algorithmic assessment (Gender API) of first-name gender probabilities resulted in 1 gender meeting or exceeding 80%.

On February 23, 2019, we captured online dissemination metrics. Every person who tweeted or retweeted a link containing the digital object identifier to each study was recorded. The gender of disseminators with active accounts on Twitter was obtained by the same mechanisms that were used for authors.

We performed χ^2^ tests for statistical comparisons. *P* values were considered significant at less than .05. Analyses were performed using R statistical software version 3.4.0 (R Project for Statistical Computing). Data were analyzed from January 11 to February 15, 2020.

## Results

We identified 39 physician compensation studies (Table 1). Among 37 unique first authors and last authors, women were listed as the first authors in 22 studies (59.5%), and last authors for 19 studies (51.4%). Among 148 unique middle authors, 82 (55.4%) were women. Among 311 identified articles citing these studies, 200 unique women (64.3%) were identified as first authors, and among 789 unique middle authors for these articles, 446 (56.5%) were women (Table 2). There was an approximately equal balance of last authors of citations, a role often denoting the senior author, including 124 unique women last authors (49.8%) and 125 unique men last authors (50.2%) with men last authors. Among 1435 disseminator tweets identified, 913 tweets (63.6%) were by women (Table 2). When our data were compared with Association of American Medical Colleges data on full-time academic women faculty of clinical departments in 2015,^6^ which suggest that 40% of full-time academic faculty are women, our data revealed significantly greater proportions of women in total authors (117 women of 209 authors [56.0%]; *P* < .001), citation authors (716 women of 1264 authors [56.6%]; *P* < .001), and Twitter disseminators (913 women of 1435 disseminators [63.6%]; *P* < .001) (Table 2).

**Table 1.** Included Physician Compensation Studies

Source	Journal	Setting	Participants	Type of compensation
Dermody et al, 2019	*Laryngoscope*	Governmental	Otolaryngologists at Veterans Affairs medical centers	Salary^a^
Guss et al, 2019	*International Journal of Radiation Oncology, Biology, Physics*	Academic	Radiation oncology faculty at US public medical schools	Salary^a^
Weng et al, 2019	*JAMA Network Open*	Mixed	Radiation oncologists listed in CMS as receiving industry funding	Industry
Wingard et al, 2019	*Journal of the National Medical Association*	Academic	Faculty at one academic health center	Salary^a^
Apaydin et al, 2018	*Journal of General Internal Medicine*	Mixed	Mixed specialty physicians	Income
Holliday et al, 2018	*Journal of Bone and Joint Surgery, American volume*	Mixed	Orthopedic surgeons who submitted Medicare claims	Medicare reimbursement
Hoops et al, 2018	*Annals of Surgery*	Academic	Surgical faculty at 1 institution	Salary^a^
Morris et al, 2018	*Annals of Surgery*	Academic	Surgeons within 1 department	Salary^a^
Muffly et al, 2018	*Obstetrics & Gynecology*	Mixed	Obstetricians and gynecologists in the US listed in CMS as receiving industry funding	Industry
Rao et al, 2018	*JAMA Network Open*	Academic	Faculty at 1 medical school	Salary^a^
Read et al, 2018	*Annals of Internal Medicine*	Mixed	Internal medicine society physician members	Income
Tringale and Hattangadi-Gluth, 2018	*JAMA Internal Medicine*	Mixed	Mixed specialty physicians listed in CMS as receiving industry funding	Industry
Trotman et al, 2018	*Open Forum Infectious Diseases*	Mixed	Infectious disease society members	Income
Weiss et al, 2018	*American Journal of Surgery*	Mixed	Mixed specialty physicians	Industry
Eloy et al, 2017	*JAMA Otolaryngology–Head & Neck Surgery*	Academic	Academic otolaryngologists listed in CMS as receiving industry funding	Industry
Kapoor et al, 2017	*American Journal of Roentgenology*	Academic	Faculty at US public medical schools	Salary^a^
Madsen et al, 2017	*Academic Emergency Medicine*	Academic	US academic emergency departments	Salary^a^
Nguyen et al, 2017	*Journal of the American Dental Association*	Mixed	Mixed specialty physicians	Income
Reddy et al, 2017	*JAMA Ophthalmology*	Mixed	Ophthalmologists who submitted Medicare claims	Medicare reimbursement
Rosenthal and Sabuco, 2017	*Psychosomatics*	Mixed	Psychosomatic medicine society members	Salary^a^
Amoli et al, 2016	*Clinical Orthopaedics and Related Research*	Mixed	North American pediatric orthopedic society members	Salary^a^
Bandari et al, 2016	*Urology Practice*	Mixed	Urologists listed in CMS as receiving industry funding	Industry
Desai et al, 2016	*Postgraduate Medical Journal*	Mixed	Mixed specialty physicians who submitted Medicare claims	Medicare reimbursement
Freund et al, 2016	*Academic Medicine*	Academic	Mixed specialty physicians at US public medical schools	Salary^a^
Jagsi et al, 2016	*Journal of the American College of Cardiology*	Mixed	Cardiologists	Salary^a^
Jena et al, 2016	*JAMA Internal Medicine*	Academic	Mixed specialty physicians at US public medical schools	Salary^a^
Ly et al, 2016	*The BMJ*	Mixed	Mixed specialty physicians	Income
Raj et al, 2016	*Academic Medicine*	Academic	Faculty at US public medical schools	NIH grant funding
Reddy et al, 2016	*JAMA Ophthalmology*	Mixed	Ophthalmologists listed in CMS as receiving industry funding	Industry
Spencer et al, 2016	*Journal of Urology*	Mixed	Urological society US members	Salary^a^
Baird et al, 2015	*Anesthesiology*	Mixed	Anesthesia society members	Salary^a^
Manahan et al, 2015	*Annals of Surgical Oncology*	Mixed	Breast surgery society members	Salary^a^
Rose et al, 2015	*PLoS One*	Mixed	Mixed specialty physicians	Industry
Weaver et al, 2015	*Journal of Hospital Medicine*	Academic	US hospitalists	Income
Wilett et al, 2015	*American Journal of Medicine*	Academic	US internal medicine program director society members	Salary^a^
Svider et al, 2014	*Journal of Surgical Education*	Academic	Principal investigators within ophthalmology departments	NIH grant funding
Eloy et al, 2013	*Otolaryngology–Head and Neck Surgery *	Academic	Principal investigators within otolaryngology departments	NIH grant funding
Jagsi et al, 2013	*Academic Medicine*	Academic	Mixed specialty physician recipients of NIH K awards	Salary^a^
Seabury et al, 2013	*JAMA Internal Medicine*	Mixed	Current Population Survey data on physicians	Income

Abbreviations: CMS, Center for Medicare & Medicaid Services; NIH, National Institutes of Health.

^a^ May include other contracted work–related compensation, such as bonuses and fee-for-service incentives.

**Table 2.** Representation of Women as Compensation Study Authors, Authors of Articles Citing Compensation Studies, and Disseminators

Category	Total, No.	No. (%)		*P* value^a^
		Women	Men	
Unique physician compensation study authors				
Total^b^	209	117 (56.0)	92 (44.0)	<.001
First	37	22 (59.5)	15 (40.5)	
Middle	148	82 (55.4)	66 (45.0)	
Last	37	19 (51.4)	18 (48.6)	
Unique citation authors				
Total^b^	1264	716 (56.6)	548 (43.4)	<.001
First	311	200 (64.3)	111 (35.7)	
Middle	789	446 (56.5)	343 (43.5)	
Last	249	124 (49.8)	125 (50.2)	
Unique Twitter disseminators	1435	913 (63.6)	522 (36.4)	<.001

^a^ *P* values indicate comparisons made to numbers of full-time academic faculty members within clinical science departments based on 2015 Association of American Medical Colleges data.

^b^ Total indicates number of authors after duplicate authors were removed.

Of 39 reports analyzed in this study, 23 (59.0%) reported no funding or no relevant funding. There were 2 instances (8.7%) of medical society support, 4 instances (17.4%) of institutional, and 1 instance (4.3%) each of regional, foundational, and organizational support; 1 instance (4.3%) was uncategorized, and 10 reports (43.5%) cited national grants, although it was not clear for some of these whether the grants supported this work or other work performed by the authors.

## Discussion

These findings suggest that women are significantly overrepresented as producers and disseminators of compensation studies that include gender data. Furthermore, most of this area of research is unfunded. These findings are important because women may be more engaged and knowledgeable about pay disparities, while men are disproportionately represented in leadership roles and better positioned to fix disparities. If women are primarily producing this mostly unfunded research, a cycle can develop in which women lose additional income (eg, clinical revenue) and do not receive appropriate academic credit for promotions (eg, grant funding). This study is limited by the accuracy of online gender information and to those studies found in our search.

In conclusion, there is an opportunity for men to more actively participate as producers and disseminators of compensation research. Future grant funding organizations should make these studies a priority.

## Notes

Supplement. **eAppendix.** Included Studies
