# Supplementary material for: Representation of Women in Authorship and Dissemination of Analyses of Physician Compensation
Source: JAMA Netw Open. 2020 Mar 20;3(3):e201330. doi: 10.1001/jamanetworkopen.2020.1330 (PMC7084168; doi:10.1001/jamanetworkopen.2020.1330)
Supplement: Supplement. — eAppendix. Included Studies [file jamanetwopen-3-e201330-s001.pdf]

# Supplementary Online Content

Larson AR, Cawcutt KA, Englander MJ, et al. Representation of women in authorship and dissemination of analyses of physician compensation. *JAMA Netw Open*. 2020;3(3):e201330. doi:10.1001/jamanetworkopen.2020.1330

**eAppendix.** Included Studies

This supplementary material has been provided by the authors to give readers additional information about their work.

© 2020 Larson AR et al. *JAMA Network Open.*

# **eAppendix.** Included Studies


1. Dermody SM, Litvack JR, Randall JA, et al. Compensation of otolaryngologists in the Veterans Health Administration: Is there a gender gap? *Laryngoscope.* 2019;129(1):113-118.
2. Guss ZD, Chen Q, Hu C, et al. Differences in physician compensation between men and women at United States public academic radiation oncology departments. *Int J Radiat Oncol Biol Phys.* 2019;103(2):314-319.
3. Weng JK, Valle LF, Nam GE, et al. Evaluation of sex distribution of industry payments among radiation oncologists. *JAMA Netw Open.* 2019;2(1):e187377.
4. Wingard D, Trejo J, Gudea M, et al. Faculty equity, diversity, culture and climate change in academic medicine: A longitudinal study. *J Natl Med Assoc.* 2019;111(1):46-53.
5. Apaydin EA, Chen PGC, Friedberg MW. Differences in physician income by gender in a multiregion study. *J Gen Intern Med.* 2018;33(9):1579-1581.
6. Holliday EB, Brady C, Pipkin WC, Somerson JS. Equal pay for equal work: Medicare procedure volume and reimbursement for male and female surgeons performing total knee and total hip arthroplasty. *J Bone Joint Surg Am.* 2018;100(4):e21.
7. Hoops HE, Brasel KJ, Dewey E, et al. Analysis of gender-based differences in surgery faculty compensation, promotion, and retention: Establishing equity. *Ann Surg.* 2018;268(3):479-487.
8. Morris M, Chen H, Heslin MJ, Krontiras H. A structured compensation plan improves but does not erase the sex pay gap in surgery. *Ann Surg.* 2018;268(3):442-448.
9. Muffly TM, Giamberardino WL, Guido J, et al. Industry payments to obstetricians and gynecologists under the sunshine act. *Obstet Gynecol.* 2018;132(1):9-17.
10. Rao AD, Nicholas SE, Hu C, et al. Association of a simulated institutional gender equity initiative with gender-based disparities in medical school faculty salaries and promotions. *JAMA Netw Open.* 2018;1(8):e186054.
11. Read S, Butkus R, Weissman A, Moyer DV. Compensation disparities by gender in internal medicine. *Ann Intern Med.* 2018;169(9):658-661.


© 2020 Larson AR et al. *JAMA Network Open.*

12. Tringale KR, Hattangadi-Gluth JA. Types and distributions of biomedical industry payments to men and women physicians by specialty, 2015. *JAMA Intern Med.* 2018;178(3):421-423.
13. Trotman R, Kim AI, MacIntyre AT, Ritter JT, et al. 2017 Infectious Diseases Society of America physician compensation survey: Results and analysis. *Open Forum Infect Dis.* 2018;5(12):ofy309.
14. Weiss A, Parina R, Tapia VJ, et al. Assessing the domino effect: Female physician industry payments fall short, parallel gender inequalities in medicine. *Am J Surg.* 2018;216(4):723-729.
15. Eloy JA, Bobian M, Svider PF, et al. Association of gender with financial relationships between industry and academic otolaryngologists. *JAMA Otolaryngol Head Neck Surg.* 2017;143(8):796-802.
16. Kapoor N, Blumenthal DM, Smith SE, et al. Sex differences in radiologist salary in U.S. public medical schools. *AJR Am J Roentgenol*. 2017;209(5):953-958.
17. Madsen TE, Linden JA, Rounds K, et al. Current status of gender and racial/ethnic disparities among academic emergency medicine physicians. *Acad Emerg Med.* 2017;24(10):1182-1192.
18. Nguyen Le TA, Lo Sasso AT, Vujcic M. Trends in the earnings gender gap among dentists, physicians, and lawyers. *J Am Dent Assoc*. 2017;148(4):257-262.
19. Reddy AK, Bounds GW, Bakri SJ, et al. Differences in clinical activity and Medicare payments for female vs male ophthalmologists. *JAMA Opthalmol.* 2017;135(3):205-213.
20. Rosenthal LJ, Sabuco JJ. Salaries in psychosomatic medicine: A cross-sectional survey of practicing physicians. *Psychosomatics.* 2017;58(1):92-94.
21. Amoli MA, Flynn JM, Edmonds EW, et al. Gender differences in pediatric orthopedics: What are the implications for the future workforce? *Clin Orthop Relat Res.* 2016;474(9):1973-1978.
22. Bandari J, Turner RM 2nd, Jacobs BL, et al. Urology payments from industry in the Sunshine Act. *Urol Pract.* 2016;3(5):332-337.
23. Desai T, Ali S, Fang X, et al. Equal work for unequal pay: the gender reimbursement gap for healthcare providers in the United States. *Postgrad Med J.* 2016;92(1092):571-575.
24. Freund KM, Raj A, Kaplan SE, et al. Inequities in academic compensation by gender: A follow-up to the national faculty survey cohort study. *Acad Med.* 2016;91(8):1068-1073.
25. Jagsi R, Biga C, Poppas A, et al. Work activities and compensation of male and female cardiologists. *J Am Coll Cardiol.* 2016;67(5):529-541.


© 2020 Larson AR et al. *JAMA Network Open.*

26. Jena AB, Olenski AR, Blumenthal DM. Sex differences in physician salary in US public medical schools. *JAMA Intern Med*. 2016;176(9):1294-1304.

27. Ly DP, Seabury SA, Jena AB. Differences in incomes of physicians in the United States by race and sex: observational study. *BMJ*. 2016;353:i2923.

28. Raj A, Carr PL, Kaplan SE, et al. Longitudinal analysis of gender differences in academic productivity among medial faculty across 24 medical schools in the United States. *Acad Med*. 2016;91(8):1074-1079.

29. Reddy AK, Bounds GW, Bakri SJ, et al. Representation of women with industry ties in ophthalmology. *JAMA Ophthalmol.* 2016;134(6):636-643.

30. Spencer ES, Deal AM, Pruthi NR, et al. Gender differences in compensation, job satisfaction, and other practice patterns in urology. *J Urol.* 2016;195(2):450-455.

31. Baird M, Daugherty L, Kumar KB, et al. Regional and gender differences and trends in the anesthesiologist workforce. *Anesthesiology*. 2015;123(5):997-1012.

32. Manahan E, Wang L, Chen S, et al. What is a breast surgeon worth? A salary survey of the American Society of Breast Surgeons. *Ann Surg Oncol.* 2015;22(10):3257-3263.

33. Rose SL, Sanghani RM, Schmidt C, et al. Gender differences in physicians‘ financial ties to industry: A study of national disclosure data. *PLoS One.* 2015;10(6):e0129197.

34. Weaver AC, Wetterneck TB, Whelan CT, et al. A matter of priorities? Exploring the persistent gender pay gap in hospital medicine. *J Hosp Med*. 2015;10(8):486-490.

35. Willett LL, Halvorsen AJ, McDonald FS, et al. Gender differences in salary of internal medicine residency directors: a national survey. *Am J Med*. 2015;128(6):659-665.

36. Svider PF, D’Aguillo CM, White PE, et al. Gender differences in successful National Institutes of Health funding in ophthalmology. *J Surg Educ.* 2014;71(5):680-688.

37. Eloy JA, Svider PF, Kovalerchik O, et al. Gender differences in successful NIH grant funding in otolaryngology. *Otolaryngol Head Neck Surg.* 2013;149(1):77-83.

38. Jagsi R, Griffith KA, Stewart A, et al. Gender differences in salary in a recent cohort of early-career physician-researchers. *Acad Med.* 2013;88(11):1689-1699.

39. Seabury SA, Chandra A, Jena AB. Trends in the earnings of male and female health care professionals in the United States, 1987 to 2010. *JAMA Intern Med.* 2013;173(18):1748-1750.

© 2020 Larson AR et al. *JAMA Network Open.*
